# Supplementary material for: Killer-Cell Immunoglobulin-like Receptor Diversity in an Admixed South American Population
Source: Cells. 2022 Sep 6;11(18):2776. doi: 10.3390/cells11182776 (PMC9496851; doi:10.3390/cells11182776)
Supplement: Supplementary file 1 [file cells-11-02776-s001.zip › cells-1871761-Supplementary Materials.pdf]

Table S1. Reference Haplotypes

		Centromere							Telomere								
**Haplotype**	**Nomenclature**	**3DL3**	**2DS2**	**2DL2**	**2DL3**	**2DP1**	**2DL1**	**3DP1**	**2DL4**	**3DL1**	**3DS1**	**2DL5**	**2DS3**	**2DS5**	**2DS4**	**2DS1**	**3DL2**
1	cA01~tA01	■			■	■	■	■	■	■					■		■
2	cA01~tB01 2DS5	■			■	■	■	■	■		■	■		■		■	■
3	cA01~tB01 2DS3	■			■	■	■	■	■		■	■	■			■	■
4	cB02~tA01	■	■	■				■	■	■					■		■
5	cB03~tA01 2DS5	■			■	■	■	■	■	■		■		■	■		■
6	cB01~tA01 2DS5	■	■	■		■	■	■	■	■		■		■	■		■
7	cB01~tA01 2DS3	■	■	■		■	■	■	■	■		■	■		■		■
8	cB02~tB01 2DS5	■	■	■				■	■		■	■		■		■	■
9	cB02~tB01 2DS3	■	■	■				■	■		■	■	■			■	■
10	cB01~tB01 2DS5	■	■	■		■	■	■	■		■	■	■	■		■	■
11	cB01~tB01 2DS3	■	■	■		■	■	■	■		■	■	■			■	■
12	cB04~tB03 2DS5	■	■	■								■		■		■	■
13	cA01~tB04	■			■	■	■	■	■		■	■	■	■		■	■
14	cB05~tB01	■	■			■	■	■	■		■	■		■		■	■
15	cA01~tB05	■			■	■	■	■	■	■	■				■		■
16	cB05~tA01	■	■			■	■	■	■	■					■		■
17	cA01~tB06	■			■	■	■	■	■	■	■	■	■		■		■
18	cB03~tA01 2DS3	■			■	■	■	■	■	■		■	■		■		■
19	cB01~tB05	■	■	■		■	■	■	■	■	■	■	■		■		■
20	cB01~tA01 2DS5 del 2DS2	■		■		■	■	■	■	■		■		■	■		■
21	cA02~tB02	■			■	■		■	■							■	■
22	cB04~tB03 2DS3	■	■	■								■	■			■	■
23	cA01~tA01 del 2DL1	■			■	■		■	■	■					■		■

Presence KIR gene is labeled as grey, while withe boxes indicate absence. Haplotypes are labeled by Vierra-Green nomenclature [35].

Table S2. Worldwide *KIR* gene frequencies

Population	n	3DL3	2DS2	2DL2	2DL3	2DL5	2DS3	3DL1	2DP1	2DL1	3DP1	2DL4	3DS1	2DS5	2DS1	2DS4	3DL2	
Medellin	161	1.0000	0.5340	0.5400	0.8880	0.6890	0.1990	0.9380	0.9500	0.9500	1.0000	1.0000	0.7140	0.5710	0.5960	0.9320	1.0000	
Bogota	119	1.0000	0.5800	0.6800	0.3100	0.7100	0.3600	0.7800	0.9300	0.9700	0.9800	0.7800	0.5100	0.6300	0.5400	0.9000	0.8800	[33]
Argentina	365	1.0000	0.5440	0.6250	0.8650	0.5570	0.2860	0.9500	0.9610	0.9610	1.0000	1.0000	0.4200	0.3540	0.4540	0.9500	1.0000	http://www.allelefrequencies.net/pop6001c.asp?pop_id=1880
BrEuropA	109	1.0000	0.3070	0.2980	0.6950	0.3730	0.1840	0.7240	N.R	0.7960	N.R	0.9770	0.2480	0.1920	0.2570	0.7350	0.9830	[78]
BrJapanA	75	0.9890	0.1110	0.1180	0.8820	0.1810	0.0550	0.8200	N.R	0.9030	N.R	0.9640	0.1320	0.1320	0.1670	0.8030	0.9730	[78]
BrParana	289	1.0000	0.4710	0.4710	0.8930	0.5260	0.2660	0.9410	0.9690	0.9720	1.0000	1.0000	0.3910	0.3460	0.4080	0.9380	1.0000	[58]
BrMatoGr	206	1.0000	0.5100	0.5100	0.8350	0.5950	0.3380	0.8820	0.9850	0.9800	1.0000	1.0000	0.4220	0.3740	0.4730	0.9320	1.0000	[61]
BrRioJan	166	0.9940	0.5660	0.5960	0.8730	0.6140	0.3130	0.9640	0.9760	0.9700	1.0000	0.9940	0.3780	0.4040	0.4160	0.9520	1.0000	http://www.allelefrequencies.net/pop6001c.asp?pop_id=2444
BrPortoA	377	1.0000	0.4160	0.4670	0.8940	0.5890	0.2280	0.9390	0.9550	0.9520	1.0000	1.0000	0.4060	0.4300	0.4320	0.9390	1.0000	[59]
BrSaoPa	289	1.0000	0.4700	0.4700	0.8900	0.5300	0.2700	0.9400	0.9700	0.9700	1.0000	1.0000	0.3900	0.3500	0.4100	0.9400	1.0000	[60]
Chile	90	1.0000	0.3890	0.3780	0.9560	0.7220	0.2890	0.8670	0.9890	1.0000	1.0000	1.0000	0.5780	0.3780	0.3440	0.8890	1.0000	[56]
MxGuer	120	1.0000	0.3800	0.3800	0.9600	0.5300	0.1300	0.9000	0.9500	0.9700	1.0000	1.0000	0.4700	0.4500	0.4900	0.9000	0.9800	[55]
MxJalis	124	1.0000	0.4600	0.4800	0.9500	0.5500	0.2200	0.9400	0.9800	0.9800	1.0000	1.0000	0.5100	0.4200	0.4500	0.9500	0.9900	[55]
Mexico	300	1.0000	0.4000	0.4070	0.9370	0.4800	0.1570	0.9430	0.9500	0.9630	1.0000	1.0000	0.4300	0.3900	0.4230	0.9230	1.0000	http://www.allelefrequencies.net/pop6001c.asp?pop_id=2835
Panama	116	1.0000	0.4740	0.4910	0.8620	0.6210	0.2500	0.9140	0.9830	0.9830	1.0000	1.0000	0.4140	0.4480	0.4400	0.9140	1.0000	http://www.allelefrequencies.net/pop6001c.asp?pop_id=3140
Uruguay	41	1.0000	0.6340	0.6330	0.9270	0.6590	0.2440	0.9510	0.9020	0.9510	1.0000	1.0000	0.5000	0.4880	0.4630	0.9510	1.0000	http://www.allelefrequencies.net/pop6001c.asp?pop_id=2435
Venezuela	205	0.9950	0.4930	0.4930	0.9320	0.5950	0.2100	0.8980	0.9560	0.9710	1.0000	1.0000	0.4340	0.4100	0.4240	0.8980	1.0000	[57]
ChinHun	448	1.0000	0.2370	0.2390	0.9870	0.3930	0.1900	0.9670	0.9960	0.9960	1.0000	1.0000	0.3260	0.2300	0.3500	0.9780	1.0000	[71]
ChinMon	160	1.0000	0.1940	0.1560	0.9880	0.4310	0.1380	0.9560	0.9880	0.9880	1.0000	1.0000	0.3690	0.3250	0.4190	0.9560	1.0000	[71]
ChinShe	503	1.0000	0.2070	0.2070	0.9840	0.3760	0.1850	0.9620	0.9920	0.9920	1.0000	1.0000	0.3360	0.2290	0.3260	0.9620	1.0000	[72]
ChinYun	404	1.0000	0.1810	0.1810	0.9980	0.3690	0.1660	0.9550	0.9950	0.9950	0.995	1.0000	0.3890	0.3020	0.3540	0.9580	1.0000	[73]
ChinZhe	273	1.0000	0.2670	0.2640	0.9930	0.4470	0.1940	0.9410	0.9960	0.9930	1.0000	1.0000	0.3960	0.2890	0.3960	0.9410	1.0000	[74]
England	584	1.0000	0.5340	0.5290	0.8970	0.5550	0.2950	0.9440	0.9620	0.9620	1.0000	1.0000	0.4440	0.3630	0.4360	0.9430	1.0000	[70]
France	108	1.0000	0.5100	0.5000	0.9100	0.4700	0.3100	0.9600	0.9700	0.9700	0.9700	1.0000	0.4400	0.2700	0.3600	0.9600	1.0000	[63]
IndianN	512	1.0000	0.5390	0.7480	0.7150	0.7380	0.4100	0.8160	0.9020	0.8570	0.9260	1.0000	0.5100	0.5780	0.5370	0.8320	1.0000	[49]
IndianO	161	1.0000	0.6580	0.6020	0.7950	0.6890	0.4350	0.8760	0.9320	0.9440	0.9940	1.0000	0.4530	0.4780	0.5220	0.8880	1.0000	[53]
IranP	248	1.0000	0.5400	0.5680	0.9100	0.5850	0.3790	0.9600	0.9800	0.9800	1.0000	1.0000	0.3300	0.2540	0.3500	0.9600	1.0000	[65]
Iran	200	1.0000	0.5750	0.5650	0.8650	0.6150	0.3800	0.9150	0.9650	0.9650	1.0000	1.0000	0.4450	0.4000	0.4550	0.9150	1.0000	http://www.allelefrequencies.net/pop6001c.asp?pop_id=2565
Ireland	240	1.0000	0.4210	0.4210	0.8920	0.4830	0.2130	0.9630	0.9580	0.9580	1.0000	1.0000	0.3960	0.3540	0.3960	0.9630	1.0000	[66]
Ireland2	200	1.0000	0.5200	0.5100	0.9300	0.4400	0.2800	0.9500	0.9700	0.9600	1.0000	1.0000	0.3500	0.2600	0.3400	0.9500	1.0000	http://www.allelefrequencies.net/pop6001c.asp?pop_id=1429
Italy	217	1.0000	0.5300	0.5300	0.8800	0.3300	0.3300	0.9600	0.9800	0.9500	0.9600	1.0000	0.3500	0.2800	0.3600	0.8900	1.0000	http://www.allelefrequencies.net/pop6001c.asp?pop_id=1987
Japan	132	1.0000	0.1140	0.1140	1.0000	0.3560	0.1360	0.9920	1.0000	1.0000	1.0000	1.0000	0.3190	0.2200	0.3330	0.9920	1.0000	[75]
Korean	159	1.0000	0.1950	0.1260	1.0000	0.3710	0.1820	0.9810	1.0000	1.0000	1.0000	1.0000	0.3520	0.2080	0.3520	0.9810	1.0000	[76]
Arabia	162	1.0000	0.5740	0.5620	0.9140	0.5620	0.3830	0.9570	0.9690	0.9630	1.0000	1.0000	0.3460	0.2780	0.3330	0.9380	1.0000	http://www.allelefrequencies.net/pop6001c.asp?pop_id=2809
Senegal	118	1.0000	0.4200	0.5500	0.9000	0.5200	0.2400	0.9900	1.0000	1.0000	1.0000	1.0000	0.0400	0.3000	0.1300	1.0000	1.0000	[63]
SouthAfr	167	1.0000	0.5990	0.6830	0.8200	0.6110	0.2630	0.9940	0.9940	0.9940	1.0000	1.0000	0.0720	0.4310	0.1260	0.9880	1.0000	[62]

Spain	339	1.0000	0.6250	0.6170	0.8290	0.5520	0.3660	0.9320	0.9500	0.9500	1.0000	1.0000	0.3920	0.3320	0.4040	0.9320	1.0000	[100]
SpainCan	230	1.0000	0.4590	0.4640	0.8880	0.5920	0.3050	0.9230	0.9830	0.9830	1.0000	0.9960	0.4640	0.3950	0.4680	0.9190	1.0000	http://www.allelefrequencies.net/pop6001c.asp?pop_id=3441
SpainSev	278	1.0000	0.5930	0.5930	0.8560	0.5470	0.3160	0.9500	0.9530	0.9530	1.0000	1.0000	0.4140	0.3560	0.4140	0.9500	1.0000	http://www.allelefrequencies.net/pop6001c.asp?pop_id=3132
Thailand	235	1.0000	0.4000	0.4040	0.9020	0.5190	0.3230	0.9490	0.9620	0.9530	1.0000	1.0000	0.4430	0.2850	0.4720	0.9400	1.0000	[69]
Turkey	154	1.0000	0.6040	0.5970	0.8570	0.5520	0.3510	0.9610	0.9610	0.9810	1.0000	1.0000	0.3250	0.2860	0.3640	0.9420	1.0000	[64]
USACa	195	1.0000	0.4970	0.4920	0.8870	0.5280	0.2820	0.9490	0.9790	0.9690	1.0000	1.0000	0.3950	0.3590	0.3740	0.9490	1.0000	[67]
USACa2	255	1.0000	0.5330	0.5290	0.8670	0.5490	0.2940	0.9490	0.9530	0.9530	1.0000	1.0000	0.3880	0.3450	0.4120	0.9490	1.0000	[68]
USAHis	128	1.0000	0.4690	0.4690	0.9380	0.6020	0.2730	0.9380	1.0000	0.9840	1.0000	1.0000	0.4530	0.4300	0.4450	0.9380	1.0000	[67]

N.R: Not Reported.

Medellin, **Bogota:** individuals from Andean and Pacific region of Colombia, **Argentina**: individuals from Buenos Aires, **BrEuropA**: Brazilians of European ancestry, **BrJapanA**: Brazilians of Japanese ancestry, **BrParana:** individuals from Parana state of southern Brazil, **BrMatoGr:** individuals from Mato Grosso state of southern Brazil, **BrRioJan:** indivuals from Rio de Janeiro, **BrPortoA:** individuals from Porto Velho of Rondonia, Brazil, **BrSaoPa;** individuals from São Paulo, **Chile:** individuals from Talca, **MxGuer:** individuals from Guerrero state, **MxJalis:** individuals from Jalisco state, **Mexico:** individuals from Zacatecas state**, Panama:** individuals from Panama city**, Uruguay:** individuals from Montevideo, **Venezuela:** individuals from Merida, **ChinHun:** individuals from Hunan province, **ChinMon:** individuals from Guangdong province, **ChinShe:** individuals from Shenzhen, **ChinYun:** individuals from Yunnan Province, **ChinZhe:** individuals from Zhejiang Province, **England:** individuals from London, Cambridge and Portsmouth, **France:** individuals from Nantes, **IndianN:** individuals from Uttar Pradesh State, **IndianO:** individuals from Bombay, **IranP:** individuals from Fars province, **Iran:** individuals from Tehran, **Ireland:** individuals from Dublin, **Ireland2:** individuals from Cookstown, **Italy:** individuals from Bologna, **Japan:** Japanese individuals in Stanford University, **Korean:** individuals from Seoul, **Arabia:** individuals from Riyadh, **Senegal:** individuals from Senegal, **SouthAfr:** individuals from Johannesburg, Tygerberg and Durban, **Spain:** individuals from Asturias autonomous community, **SpainCan:** individuals from Cantabria autonomous community, **SpainSev:** individuals from Sevilla, **Thailand:** individuals from Khon Kaen province, **Turkey:** individuals from Istanbul, **USACa:** Caucasian individuals from United States in European Region, **USACa2:** Caucasian individuals from Los Angeles of United States, **USAHis:** Hispanic individuals from Los Angeles of United States.

Table S3. Amerindians *KIR* gene frequencies.

Population	n	3DL3	2DS2	2DL2	2DL3	2DL5	2DS3	3DL1	2DL1	2DL4	3DS1	2DS5	2DS1	2DS4	3DL2	
Medellin	161	1.0000	0.5340	0.5400	0.8880	0.6890	0.1990	0.9380	0.9500	1.0000	0.7140	0.5710	0.5960	0.9320	1.0000	
Bogota	119	1.0000	0.5800	0.6800	0.3100	0.7100	0.3600	0.7800	0.9700	0.7800	0.5100	0.6300	0.5400	0.9000	0.8800	[33]
ArgWichi	101	1.0000	0.6110	0.6200	0.8400	0.5300	0.0300	0.8900	0.8400	1.0000	0.5400	0.5200	0.5300	0.8900	1.0000	[52]
ArgChirig	54	1.0000	0.4100	0.4400	0.8700	0.5900	0.0600	0.8700	0.9100	1.0000	0.5700	0.5600	0.5700	0.8700	1.0000	[52]
BrGuarKai	150	1.0000	0.1100	0.1090	0.8890	0.4790	0.0130	0.5130	0.8930	0.9970	0.4830	0.4770	0.4840	0.5100	0.9980	[78]
BrGuarÑan	81	0.9990	0.1560	0.1560	0.8440	0.3940	0.0060	0.6050	0.8490	0.9930	0.3880	0.3880	0.3930	0.6060	0.9980	[78]
BrGuarMby	84	1.0000	0.3930	0.3930	0.5950	0.5060	0.0950	0.4710	0.6010	0.9990	0.5300	0.4230	0.5180	0.4760	0.9950	[78]
BrKaingIv	93	0.9990	0.3060	0.3160	0.6720	0.3980	0.0000	0.6030	0.6980	0.9900	0.3820	0.4020	0.3930	0.6030	0.9940	[78]
BrKaingCo	64	1.0000	0.3440	0.3520	0.6490	0.4930	0.0000	0.5000	0.6490	0.9930	0.4840	0.4920	0.4930	0.4930	1.0000	[78]
MxHuichol	73	1.0000	0.3400	0.3400	1.0000	0.5600	0.1600	0.9700	1.0000	1.0000	0.5600	0.4800	0.5600	0.9700	1.0000	[77]
MxPurepac	53	1.0000	0.3400	0.3400	1.0000	0.6200	0.0400	0.9800	1.0000	1.0000	0.6200	0.6200	0.6200	0.9800	1.0000	[77]
MxTarahum	65	1.0000	0.3400	0.3400	1.0000	0.6600	0.0000	0.9850	1.0000	1.0000	0.6600	0.6600	0.6600	0.9850	1.0000	[77]
ParagAche	51	0.9900	0.0100	0.0100	0.9900	0.3730	0.0100	0.6080	1.0000	1.0000	0.3920	0.3920	0.3820	0.6080	1.0000	[78]
VenBari	80	1.0000	0.4375	0.4375	0.8875	0.6375	0.0000	0.8250	0.8875	1.0000	0.6125	0.6375	0.6375	0.8125	1.0000	[50]
VenWarao	89	1.0000	0.5725	0.5953	0.9548	0.6740	0.2021	0.8203	0.9438	1.0000	0.5843	0.5618	0.6854	0.8203	1.0000	[50]
VenYucpa	61	1.0000	0.7050	0.7050	0.7050	0.7378	0.0000	0.7049	0.7049	1.0000	0.7377	0.7377	0.7377	0.7049	1.0000	[51]

Medellin, **Bogota:** individuals from Andean and Pacific region of Colombia, **ArgWichi**: Wichis from Chaco and Salta province, **ArgChirig**: Chiriguanos from Salta province, **BrGuarKai**: Guarani Kaiowa, **BrGuarÑan**: Guarani Nandeva, **BrGuarMby**: Guarani Mbya, **BrKaingIv**: Kaingang from Iva, **BrKaingCo**: Kaingang from Rio das Cobras, **MxHuichol**:Huicholes from Sierra Madre Occidental, **MxPurepac**: Purepechas from northwestern region of Michoacán, **MxTarahum**: Tarahumaras from Chihuahua, **ParagAche**: Aches from Paraguay, **VenBari**: Bari tribe from Venezuela, **VenWarao**: Warao tribe from northeastern Venezuela, **VenYucpa**: Yucpa from Serranía del Perijá, Venezuela.

Table S4. The most common estimated *KIR* haplotypes in Amerindians.

	Centromere							Telomere										Venezuela [50,51]			Mexico [77]			Argentina [52]		Brazil [78]					
**Haplotype Nomenclature**	3DL3	2DS2	2DL2	2DL3	2DP1	2DL1	3DP1	2DL4	3DL1	3DS1	2DL5	2DS3	2DS5	2DS4	2DS1	3DL2	Medellín, (n=161) % (Colombia)	Bari (n=80) %	Warao (n=89) %	Yucpa (n=61) %	Huichol (n=73) %	Purepecha (n=53) %	Tarahumara (n=65) %	Wichi (Chaco) (n=101) %	Chiriguano (n=54) %	Guarani Kaiowá (n=150) %	Guarani Ñandeva (n=81) %	Guarani M'Byá (n=84) %	Kaingang – Ivaí (n=93) %	Kaingang - Rio das Cobras (n=64) %	Aché (n= 51) % (Paraguay)
cA01~tA01																	41.6	38.8	53.9	45.9	69	65.4	58.3	55.8	49.4	48.2	58.2	34.7	56.5	46.1	59.8
cA01~tB01 2DS5																	16.5	32.5	9	4.1	7.7	15.7	24	2.8	8.9	40.1	24.1	20.4	9.8	18	36.7
cB02~tB01																	10.9	5.6	23.6	47.5	5.4	6.3	4.1	25.3	10.8	8.1	14.6	31.1	25.5	30.5	
cB02~tA01																	9.9	19.4	0.6	2.5	1.5	2.5	7.9	11.3	8	2		7.2	1.1	3.1	
cA01~tB05																	5.9														
cB01~tA01 2DS3																	5										0.6				
cB01~tB01 2DS3																	2.5									0.3					1
Unresolved																	7.7	3.7	7.9	0	2.7	1.9	1.5	3.7	18.5	0.3	0.6	0.6	4.9	0.8	2

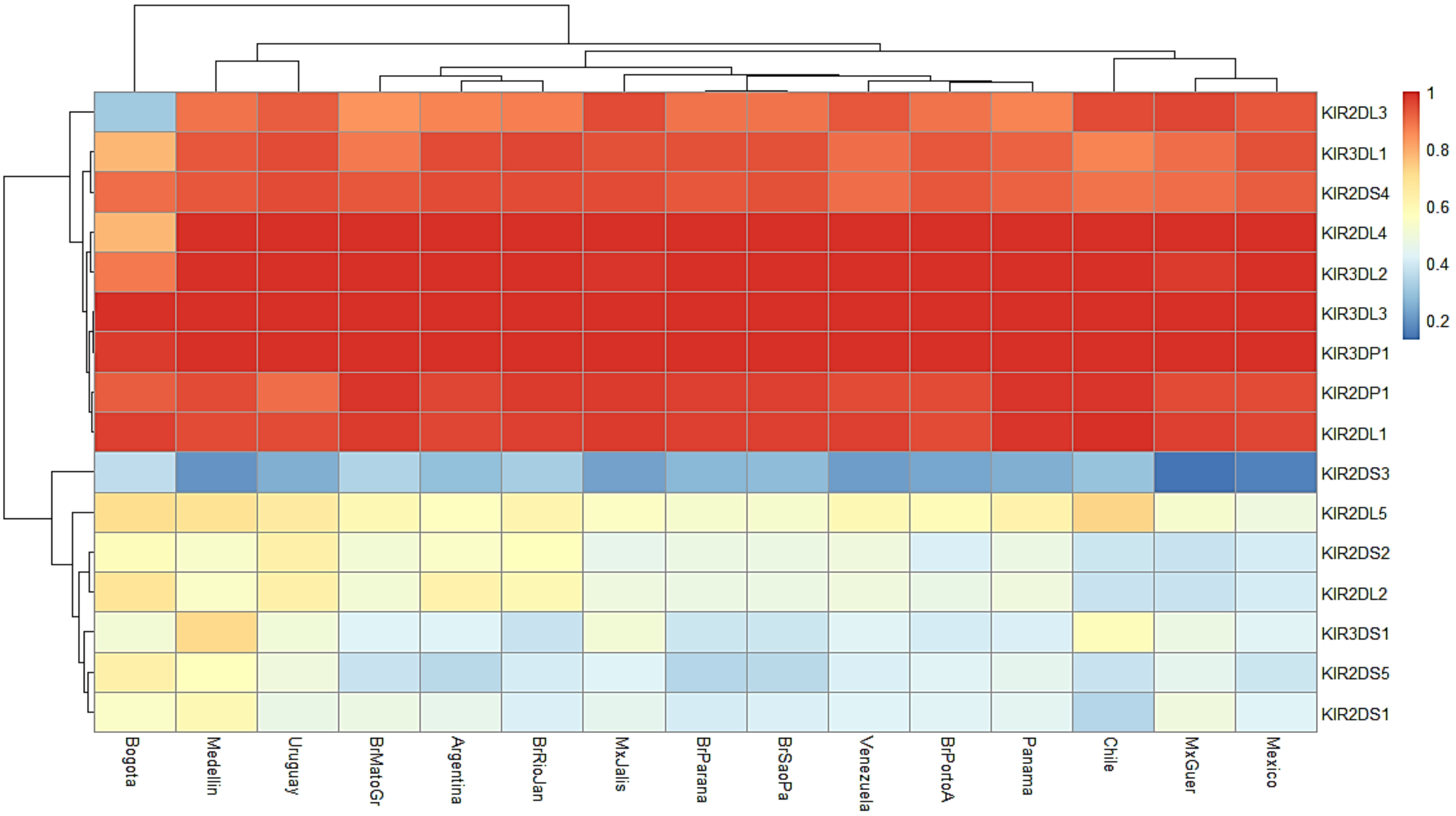

Figure S1. Heatmap for *KIR* gene frequencies from South and Central American populations. The heatmap was constructed with 15 American populations from Brazil (n=1327), Mexico (n=544), Argentina (n=365), Venezuela (n=205), Panama (n=116), Chile (n=90), Uruguay (n=41), Bogota - Colombia (n=119) and Medellin – Colombia (n=161). The deeper color indicated higher observed carrier frequency from 0 to 1.

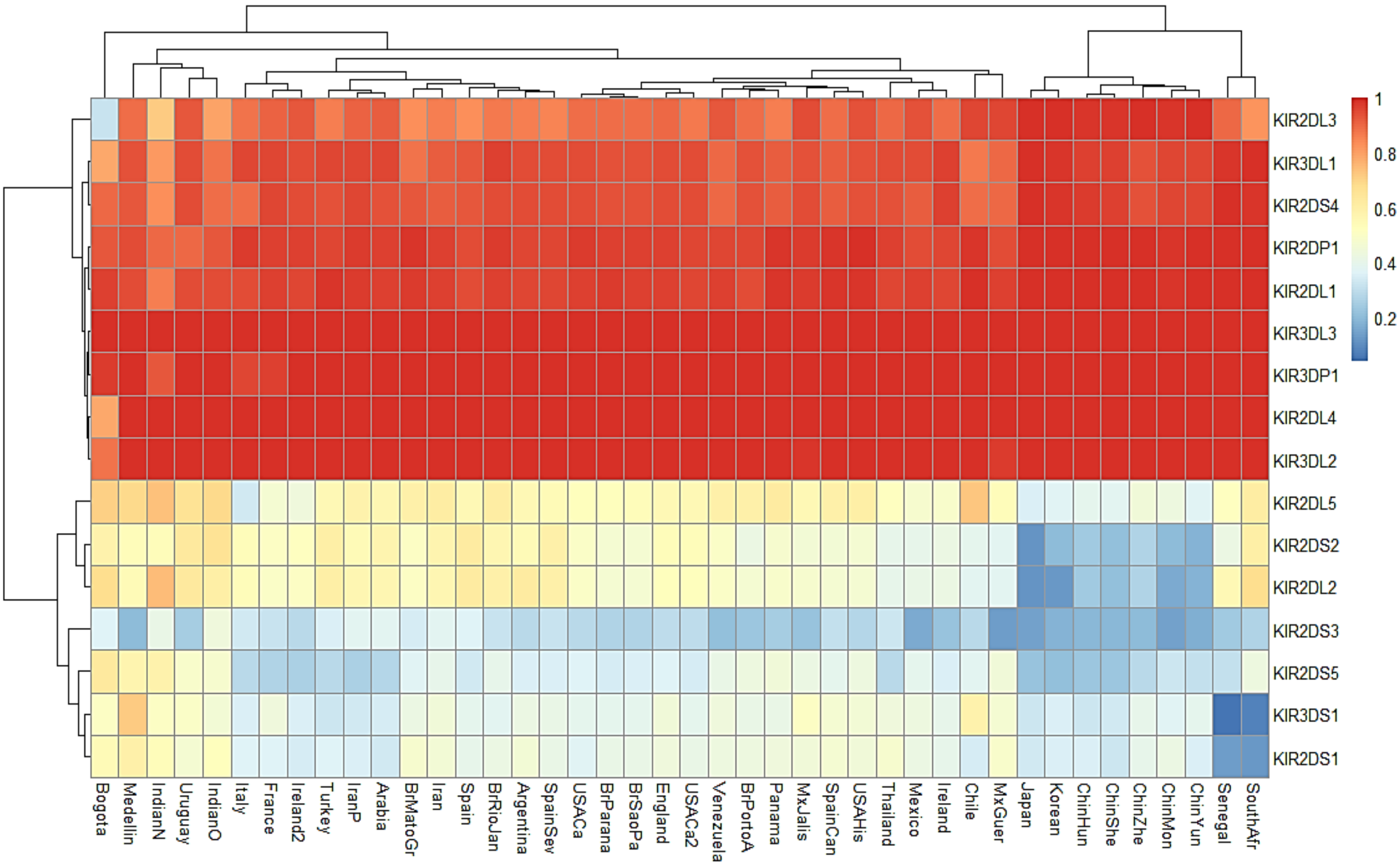

Figure S2. Heatmap for *KIR* gene frequencies from worldwide populations. The heatmap was constructed with 42 populations from China (n=1788), Brazil (n=1327), Spain (n=847), India (n=673), England (n=584), United States (n=578), Mexico (n=544), Iran (n=448), Ireland (n=440), Argentina (n=365), Thailand (n=235), Italy (n=237), Venezuela (n=205), South Africa (n=167), Arabia (n=162), South Korea (n=159), Turkey (n=154), Japan (n=132), Senegal (n=118), Panama (n=116), France (n=108), Chile (n=90), Uruguay (n=41), Bogota - Colombia (n=119) and Medellin – Colombia (n=161). The deeper color indicated higher observed carrier frequency from 0 to 1.

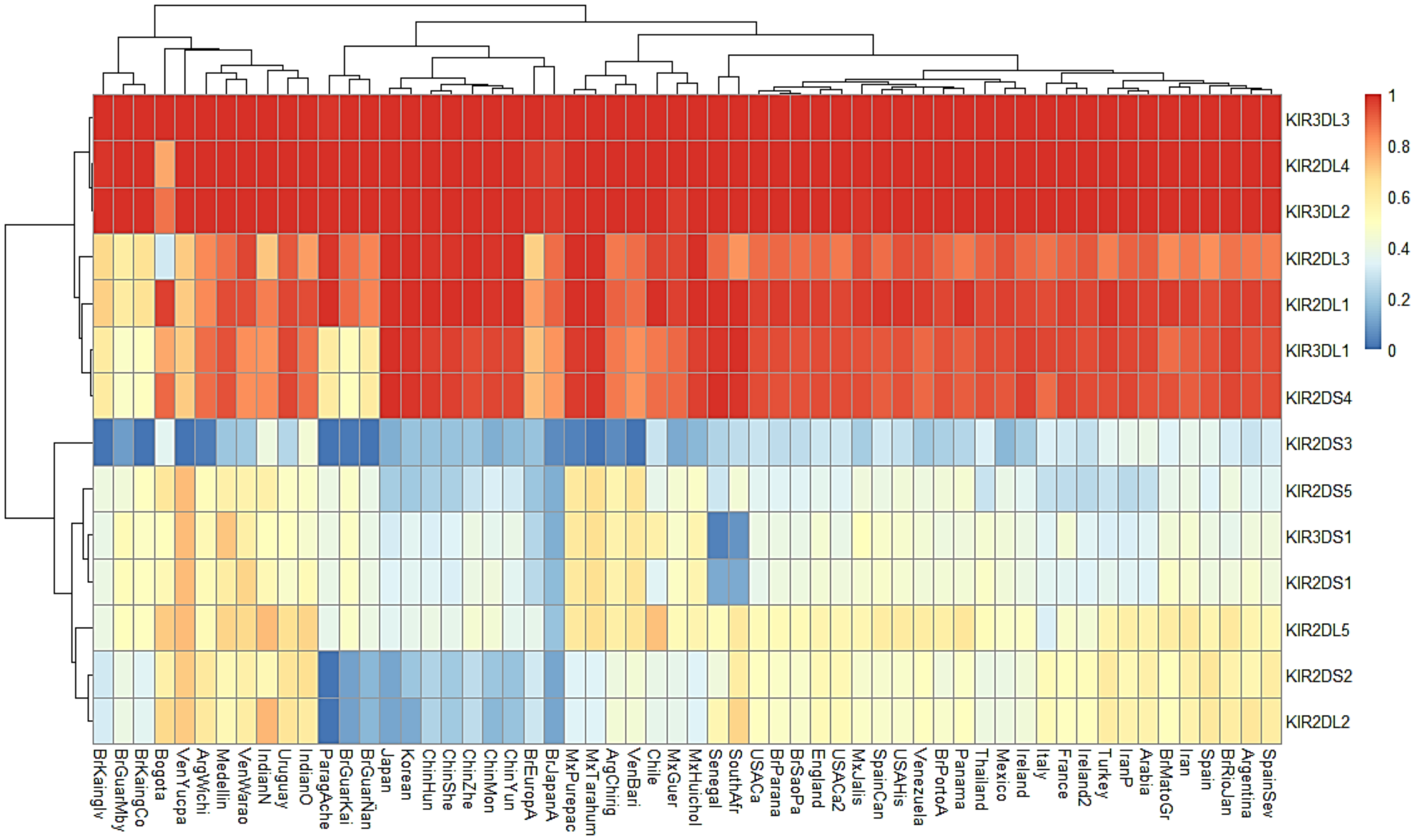

Figure S3. Heatmap for *KIR* gene frequencies from worldwide populations, including Amerindians. The heatmap was constructed with 42 populations from China (n=1788), Brazil (n=1327), Spain (n=847), India (n=673), England (n=584), United States (n=578), Mexico (n=544), Iran (n=448), Ireland (n=440), Argentina (n=365), Thailand (n=235), Italy (n=237), Venezuela (n=205), South Africa (n=167), Arabia (n=162), South Korea (n=159), Turkey (n=154), Japan (n=132), Senegal (n=118), Panama (n=116), France (n=108), Chile (n=90), Uruguay (n=41), Bogota - Colombia (n=119) and Medellin – Colombia (n=161). Furthermore, we included 16 Amerindian populations from Brazil (n=656), Venezuela (n=230), Mexico (n=191), Argentina (n=155), and Paraguay (n=51). The deeper color indicated higher observed carrier frequency from 0 to 1.

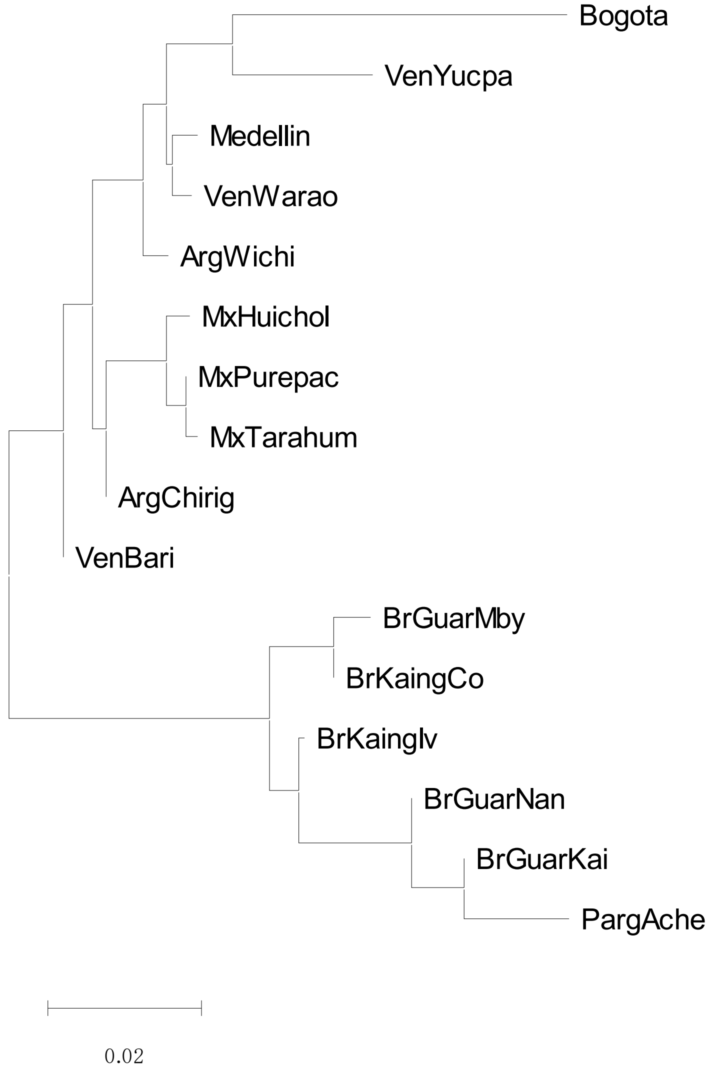

Figure S4. Neighbor-joining (NJ) dendrogram of Nei´s genetic distances among Amerindian populations, based on the KIR genes frequencies. The NJ dendrogram was constructed with 16 Amerindian populations from Brazil (n=472), Venezuela (n=230), Mexico (n=191), Argentina (n=155), and Paraguay (n=51). Furthermore, we include Bogota - Colombia (n=119) and Medellin – Colombia (n=161) populations. The bar suggests a 0.02 (20%) *KIR* gene frequencies variation for the length of the scale.

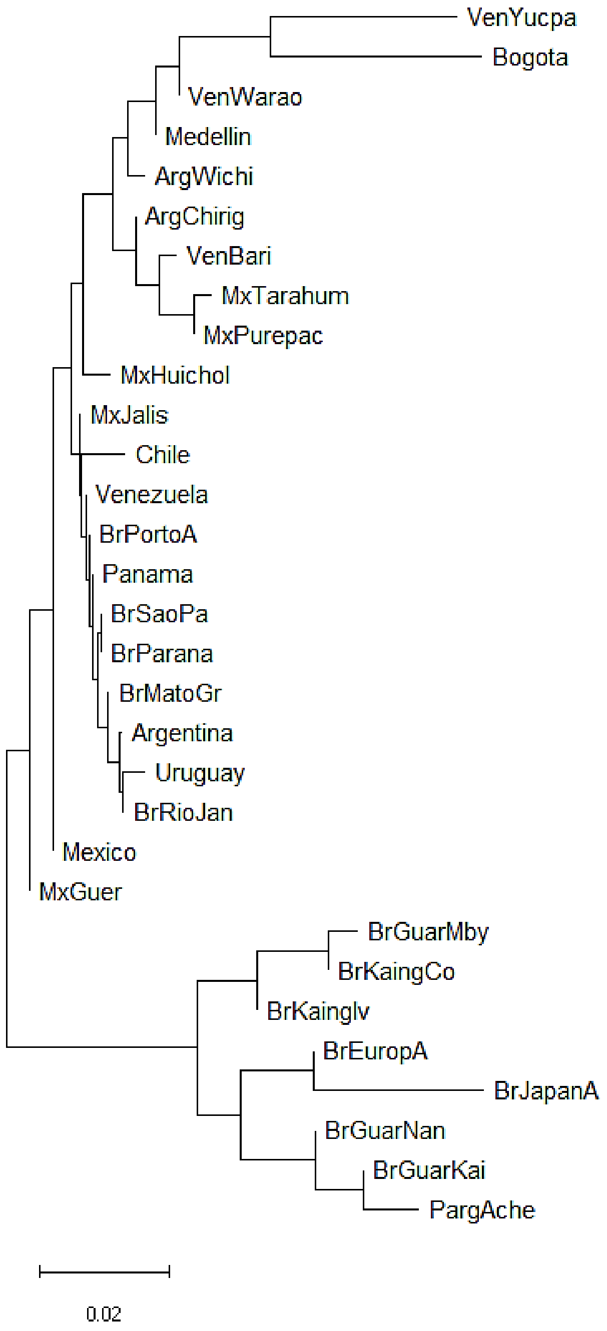

Figure S5. Neighbor-joining (NJ) dendrogram of Nei´s genetic distances among Amerindian, Central and South American populations, based on the KIR genes frequencies. The NJ dendrogram was constructed with 15 American populations from Brazil (n=1327), Mexico (n=544), Argentina (n=365), Venezuela (n=205), Panama (n=116), Chile (n=90), Uruguay (n=41), Bogota - Colombia (n=119) and Medellin – Colombia (n=161) and 16 Amerindian populations from Brazil (n=656), Venezuela (n=230), Mexico (n=191), Argentina (n=155), and Paraguay (n=51). The bar suggests a 0.02 (20%) *KIR* gene frequencies variation for the length of the scale.

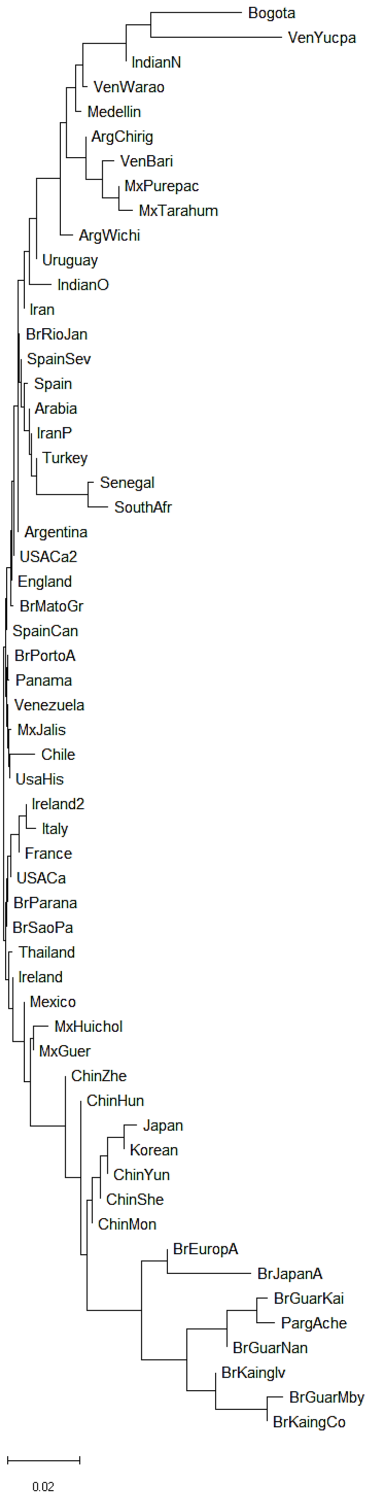

Figure S6. Neighbor-joining (NJ) dendrogram of Nei´s genetic distances among worldwide populations, based on the KIR genes frequencies. The NJ dendrogram was constructed with 42 populations from China (n=1788), Brazil (n=1327), Spain (n=847), India (n=673), England (n=584), United States (n=578), Mexico (n=544), Iran (n=448), Ireland (n=440), Argentina (n=365), Thailand (n=235), Italy (n=237), Venezuela (n=205), South Africa (n=167), Arabia (n=162), South Korea (n=159), Turkey (n=154), Japan (n=132), Senegal (n=118), Panama (n=116), France (n=108), Chile (n=90), Uruguay (n=41), Bogota - Colombia (n=119) and Medellin – Colombia (n=161). Furthermore, we included 16 Amerindian populations from Brazil (n=656), Venezuela (n=230), Mexico (n=191), Argentina (n=155), and Paraguay (n=51). The bar suggests a 0.02 (20%) *KIR* gene frequencies variation for the length of the scale.

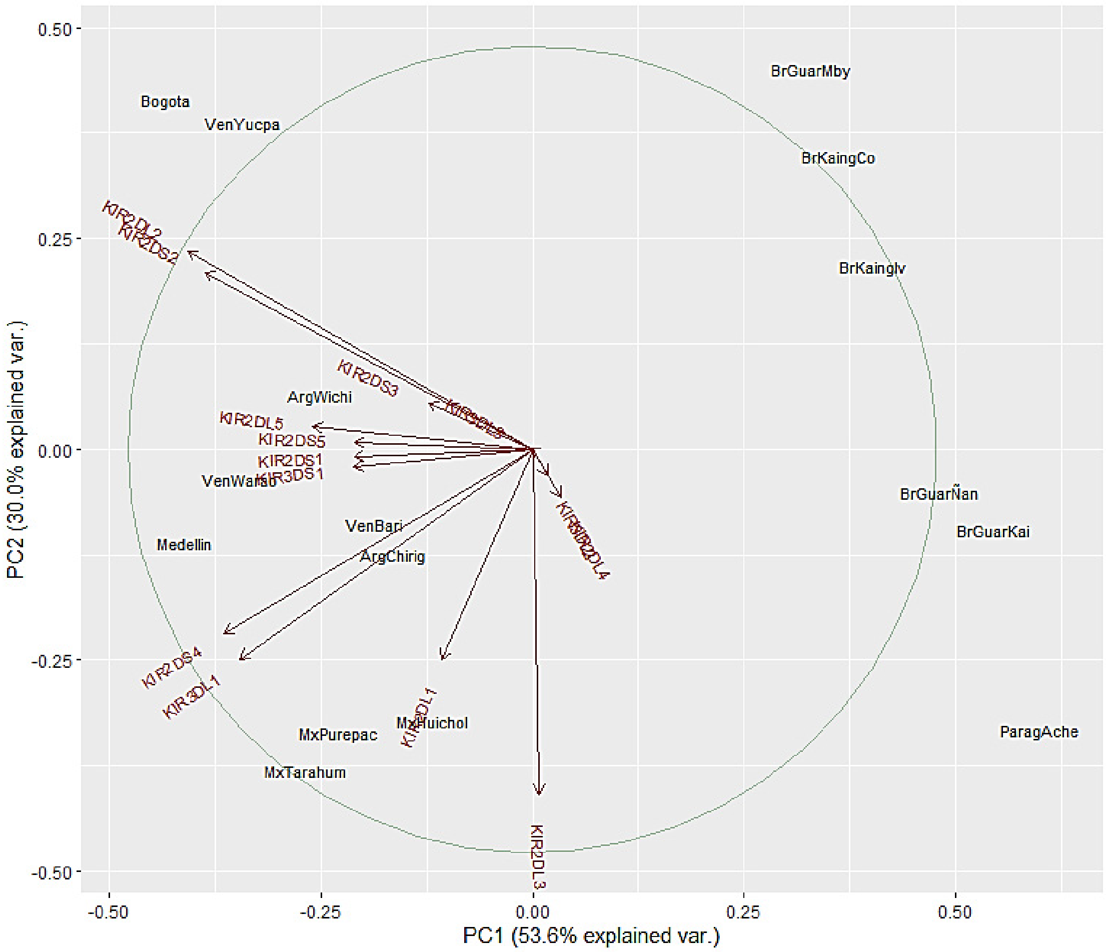

Figure S7. Principal component analysis based on 14 *KIR* genes for Amerindian populations. The circle indicates the correlation between *KIR* gene frequencies. Red arrows show the relative contribution of each *KIR* gene frequency to the variability along the first two axes (PC1 and PC2). PC1 was strongly correlated with *KIR2DL2, KIR2DS2, KIR2DL5, KIR2DS5, KIR3DS1* and *KIR2DS1* genes and PC2 was strongly correlated with *KIR2DL3, KIR3DL1* and *KIR2DS4* genes.

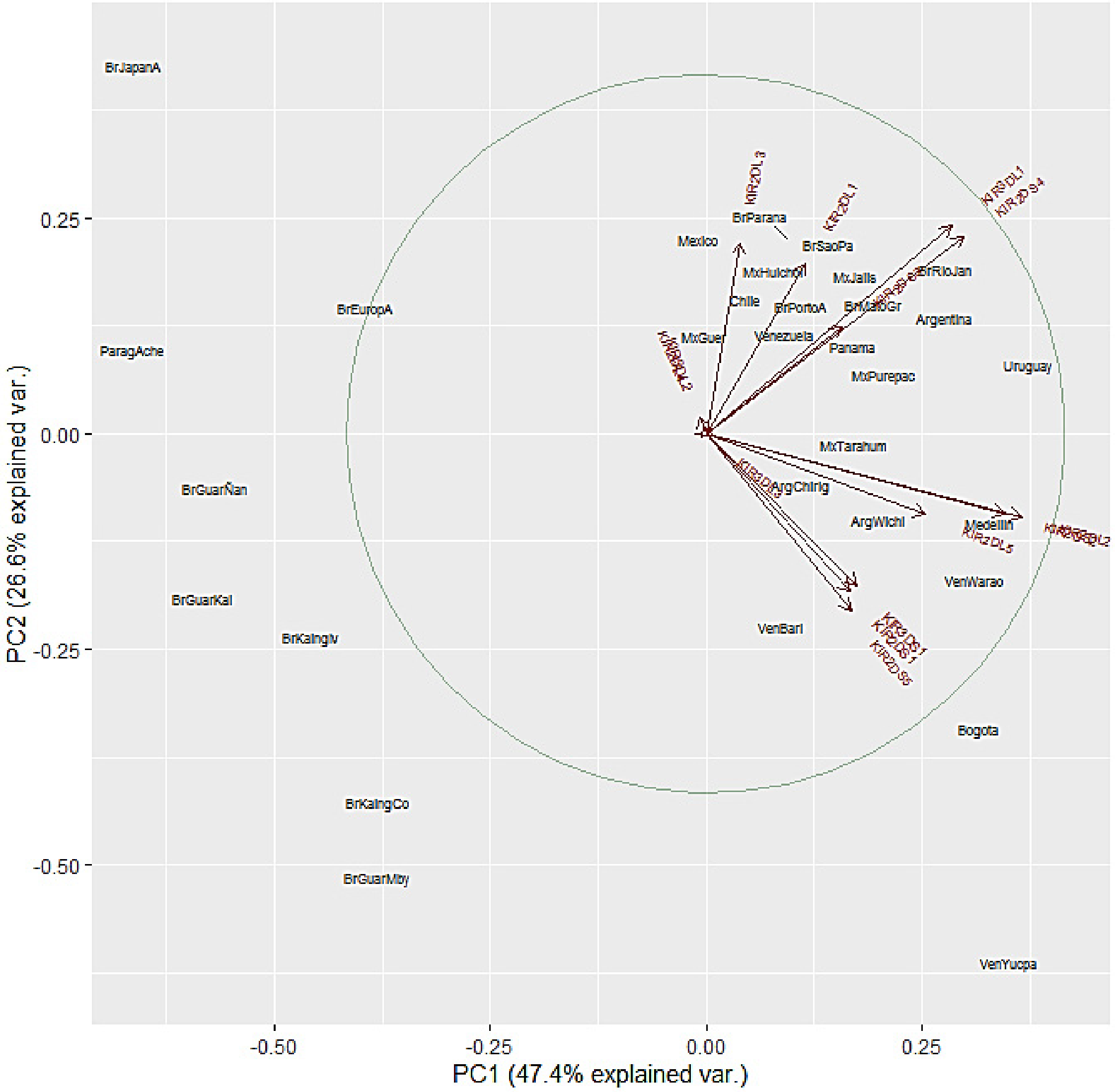

Figure S8. Principal component analysis based on 14 *KIR* genes for Amerindian, Central and South American populations. The circle indicates the correlation between *KIR* gene frequencies. Red arrows show the relative contribution of each *KIR* gene frequency to the variability along the first two axes (PC1 and PC2). PC1 was strongly correlated with *KIR2DL2* and *KIR2DS2* genes and PC2 was strongly correlated with *KIR3DL1, KIR2DS4*, *KIR2DL3*, *KIR2DS5* and *KIR2DL1* genes.

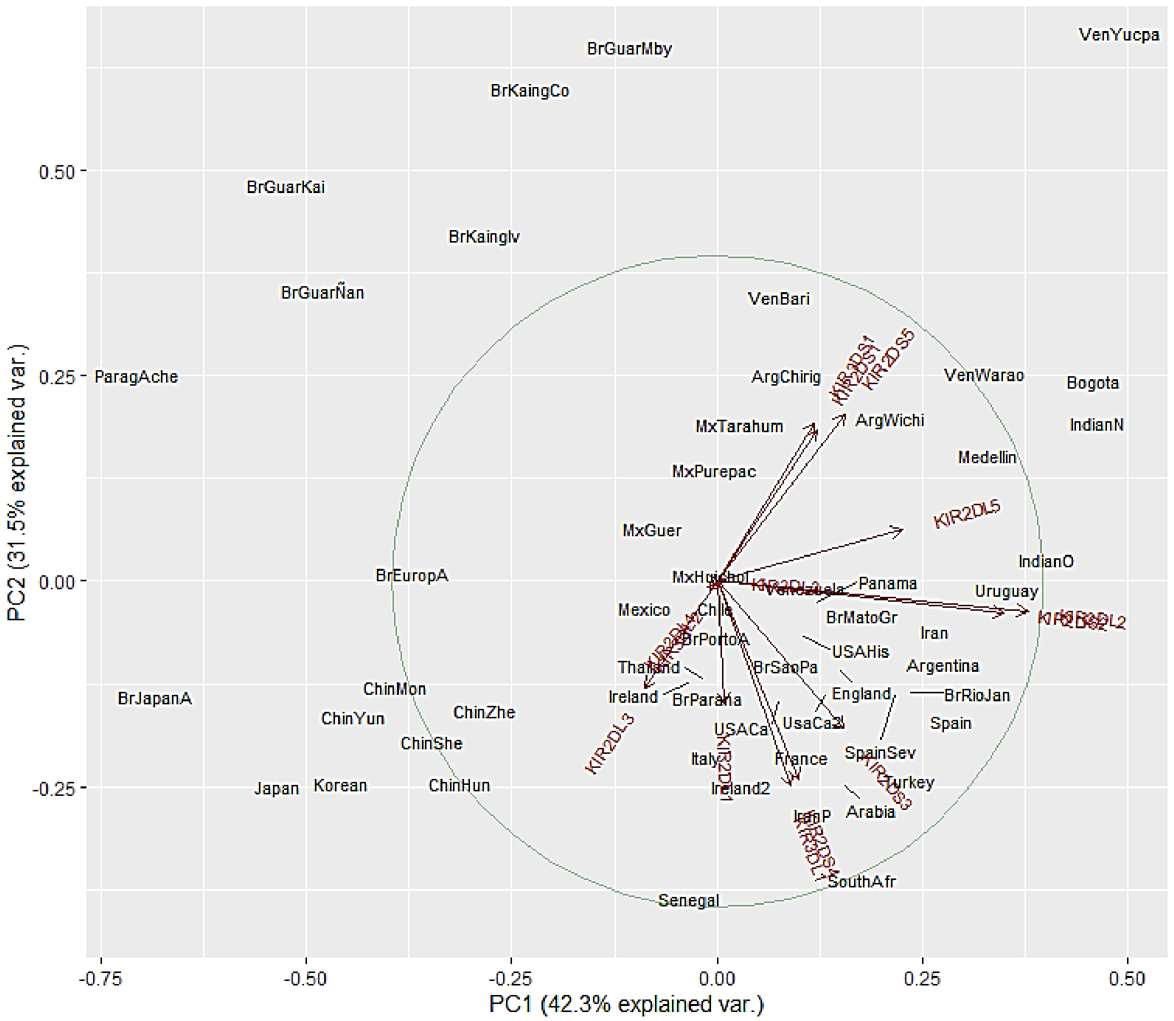

Figure S9. Principal component analysis based on 14 *KIR* genes for worldwide populations, including Amerindians. The circle indicates the correlation between *KIR* gene frequencies. Red arrows show the relative contribution of each *KIR* gene frequency to the variability along the first two axes (PC1 and PC2). PC1 was strongly correlated with *KIR2DL2* and *KIR2DS2* genes and PC2 was strongly correlated with *KIR3DL1*, *KIR2DS4*, *KIR2DS5*, *KIR3DS1* and *KIR2DS1* genes.
